# Suppression of tubulin detyrosination by parthenolide recruits the plant-specific kinesin KCH to cortical microtubules

**DOI:** 10.1093/jxb/erv012

**Published:** 2015-03-16

**Authors:** Natalie Schneider, Holger Ludwig, Peter Nick

**Affiliations:** Botanical Institute, Molecular Cell Biology, Karlsruhe Institute of Technology, Kaiserstraße 2, D-76131 Karlsruhe, Germany

**Keywords:** KCH kinesin, microtubules, parthenolide, tobacco BY-2, tubulin detyrosination.

## Abstract

Detyrosination of α-tubulin seems to be conserved in all eukaryotes. However, its biological function in plants has remained obscure. A conserved C-terminal tyrosine is removed by a still unidentified tubulin–tyrosine carboxypeptidase (TTC) and can be religated by a tubulin–tyrosine ligase (TTL). To obtain insight into the still elusive biological function of this detyrosination–tyrosination cycle, the effects of the TTC inhibitor parthenolide were analysed in BY-2 tobacco cells. Parthenolide caused a depletion of detyrosinated α-tubulin, whereas the level of tyrosinated tubulin was elevated. This biochemical effect was accompanied by growth inhibition in cycling BY-2 cells and alteration of microtubule-dependent events that define division and expansion geometry such as cell plate alignment or axial expansion. Furthermore, parthenolide triggered an apoplastic alkalinization indicative of activation of defence-related calcium influx channels. At the same time, parthenolide promoted the association of the plant-specific kinesin KCH with cortical microtubules. These observations are integrated into a working model, where detyrosination acts as signal to modulate the binding of kinesin motors involved in structural and sensory functions of the microtubular cytoskeleton.

## Highlight

By interfering with tubulin detyrosination via parthenolide treatment, the affinity of the plant-specific kinesin KCH towards microtubules is increased and results in alteration of microtubule-dependent events such as division geometry.

## Introduction

Microtubules (MTs), as a central component of the eukaryotic cytoskeleton, perform numerous different functions. These functions can be of a structural nature, such as the establishment of the mitotic spindle, as well as the regulation of cell expansion by control of cell wall texture (for a recent review, see Nick, 2014). However, they can also be related to sensing, such as the sensing of gravity, the response to osmotic stress, or the mechanical integration of plant tissues (reviewed in Nick, 2013). Whereas the building blocks of MTs, α- and β-tubulin, are mostly encoded by single genes in algae, they form small gene families in land plants. Their products, however, are relatively conserved in terms of protein sequence. In the mosses, the difference between the individual genes is mostly confined to the regulatory regions (Jost *et al.*, 2004), indicating that it was a regulatory rather than functional diversification that drove the formation of these gene families. In fact, MTs were found to be composed of different isotypes (Hussey *et al.*, 1987). This leads to the question of how MTs can be assigned to functionally different arrays.

MTs can be modified by so-called post-translational modifications (PTMs), which seem to increase functional diversity in the highly conserved MT population and also can influence the stability and structure of MTs. Numerous modifications are known (Hammond *et al.*, 2008) and have been proposed to act as a code for functional assignment that is read by different binding proteins (Parrotta *et al.*, 2014). In fact, changes of cyclic detyrosination–tyrosination as the most prominent PTM has been shown to correlate with the reorientation of cortical MTs, a key regulator of axial cell expansion in plants (Wiesler *et al.*, 2002).

The α-tubulin of almost all eukaryotic organisms harbours a C-terminal tyrosine that can be cleaved off by a tubulin–tyrosine carboxypeptidase (TTC) (MacRae, 1997). The antagonist of this TTC is a tubulin–tyrosine ligase (TTL), which can religate a tyrosine to detyrosinated tubulin. The conservation of the C-terminal tyrosine throughout eukaryotes indicates that this cycle is highly conserved. However, little is known about its function. While the TTL preferentially uses tubulin dimers as a substrate (Burns, 1987), detyrosination is mainly targeted to the polymerized form (Gundersen *et al.*, 1987). *In vivo*, both, detyrosinated and tyrosinated tubulins co-exist, whereby under normal conditions the tyrosinated form dominates (Gundersen *et al.*, 1987). In tumour cells, however, the abundance of tyrosinated tubulin is decreased, and suppression of TTL activity promotes tumour aggressiveness (Lafanechere *et al.*, 1998). Therefore, pharmacological inhibition of TTC activity represents an important target in the screening of anticancer drugs. Such inhibitors, by restoring the equilibrium between tyrosinated and detyrosinated α-tubulin, are expected to be endowed with antitumour activity. In fact, the sesquiterpene parthenolide, isolated from *Tanacetum parthenium* (Feverfew, Asteraceae), has been isolated as a TTC inhibitor during a search for anticancerogenic substances (Fonrose *et al.*, 2007).

Although the TTL has been purified and cloned from porcine brain (Ersfeld *et al.*, 1993), the enzyme responsible for the TTC activity has remained enigmatic so far. Nevertheless, it is possible to address its biological function by feeding 3-nitro-l-tyrosine (NO_2_Tyr). This compound is formed as a stress metabolite in several organisms and has been linked with the genesis of certain tumours (for a review, see Beckman and Koppenol, 1996). TTL does accept NO_2_Tyr as a substrate and integrates the nitrosylated tyrosine residue into α-tubulin, but the nitrotyrosinated tubulin seems not to be accepted by the TTC as a substrate (Chang *et al.*, 2002), such that the dominance of tyrosinated tubulin over the detyrosinated form is enhanced. This strategy was adopted in a previous work in order to address the cellular functions of the tyrosination–detyrosination cycle in plants (Jovanovic *et al.*, 2010), and dose-dependent inhibition of mitosis by NO_2_Tyr was observed in both tobacco BY-2 cells and the root meristem of rice. In addition, the sensitivity to oryzalin was increased, indicative of an elevated dynamicity of the MTs containing NO_2_Tyr. In the rapidly cycling tobacco BY-2 cells, a distorted orientation of the cross walls was also observed, suggesting that the organization of the microtubular phragmoplast was affected.

Although these cellular effects were shown to be specific (for instance, they could not be evoked by non-nitrosylated tyrosine), it remained unclear whether they are caused by the depletion of the detyrosinated form of α-tubulin or rather by the excessive formation of nitrotyrosinated α-tubulin. In the current work, an alternative approach was therefore used to inhibit TTC activity, making use of parthenolide. To the authors’ knowledge, there has been no record so far of the effect of parthenolide on plant cells. It could be shown for BY-2 cells that parthenolide can deplete the detyrosinated form of α-tubulin, and interfere with cell proliferation in a dose-dependent manner.

As a possible target for the detyrosination signal, kinesin motors have been proposed. However, it seems to depend on the specific type of kinesin as to whether detyrosination acts as a recruiting signal (Liao and Gundersen, 1998; Zekert and Fischer, 2009; Parrotta *et al.*, 2014). Some kinesins are not affected by detyrosination of their target (Zekert and Fischer, 2009), and some kinesins, such as the mammalian mitotic centromere-associated kinesin (MCAK), are even inhibited by detyrosination (Peris *et al.*, 2009).

The present analysis was therefore focused on cellular functions that are linked to the function of kinesin KCH. This plant-specific member of the kinesin-14 family links MTs and actin filaments, and occurs in two subsets that confer two different cellular functions (Klotz and Nick, 2012)—one subset is linked to the perinuclear actin basket, and is important for pre-imitotic nuclear migration and mitosis (Frey *et al.*, 2010). The second subset is not linked to actin filaments and moves slowly with cortical MTs and thus plays a role in cell expansion. In fact, it could be shown that the inhibition of proliferation by parthenolide is linked to a specific delay of pre-mitotic nuclear positioning and a disorientation of the cross walls (congruent with the effect of NO_2_Tyr) that is heralded by a disturbed geometry of the metaphase plate. In addition, it could be shown that parthenolide can activate a rapid apoplastic alkalinization indicative of calcium influx activity at the plasma membrane as well as a partial loss of MT orientation and cell axiality. Using a transgenic cell line, where KCH is tagged by green fluorescent protein (GFP), it could be observed that parthenolide enhances the interaction of KCH with cortical MTs. A working model, where tubulin detyrosination modulates (in the case of KCH: constrains) the binding of specific kinesins that convey architectural (nuclear positioning, deposition of the cell plate) and sensory (calcium channels) functions, is proposed.

## Materials and methods

### Cell lines and cell culture

Tobacco BY-2 cells (*Nicotiana tabacum* L. cv. Bright Yellow 2; Nagata *et al.*, 1992) were cultivated in Murashige and Skoog (MS) medium [4.3g l^–1^ Murashige and Skoog salts (Duchefa, Haarlem, The Netherlands), 30g l^–1^ sucrose, 200mg l^–1^ KH_2_PO_4_, 100mg l^–1^ inositol, 1mg l^–1^ thiamine, and 0.2mg l^–1^ 2,4-D, pH 5.8]. After 7 d, 1–2ml of the old cell cultures were transferred into fresh medium and kept in darkness on a rotary shaker (150rpm) at 25 °C. In addition to non-transformed cells, a transgenic line expressing the β-tubulin AtTUB6 from *Arabidopsis thaliana* fused to GFP driven by the *Cauliflower mosaic virus* (CaMV) 35S promotor (Hohenberger *et al.* 2011) was used to follow MTs *in vivo*. A second transgenic line expressing the kinesin KCH from *Oryza sativa* fused to GFP was used to visualize the effect of parthenolide on the binding of KCH to microtubules. In the case of the transgenic lines, the medium was complemented with 50mg l^–1^ kanamycin.

### Quantification of the cellular response to parthenolide

Parthenolide (≥90%, Sigma-Aldrich, Munich, Germany) was added to the cells at the time of subcultivation, and the cellular responses were quantified as described in Kühn *et al*. (2013). Culture growth was monitored based on packed cell volume. Data represent the mean and standard errors (SEs) from three independent experimental series. Nuclear positioning was measured at day 1 after subcultivation according to Frey *et al.* (2010). Data show the mean and SEs from 350 individual cells collected in three independent experimental series.

To estimate the average length of the cell cycle, *k* (Fig. 4D), the total cell number was scored over the first 3 d of cultivation (i.e. the entire cycling phase). Based on the model of exponential cell growth with *N*_*t*_=*N*_0_×e^*kt*^ (with *t*=time after subcultivation in days, *N*_0_ the initial cell number, and *k* the average length of cell the cycle, the values for *k* could be fitted using a linear regression of ln (*N*_t_) over time with very tight correlations (*r* >0.95).

The effect of parthenolide on the orientation of cross walls was quantified as described in Jovanovic *et al.* (2010) as the ratio of the angles between the cross wall and the side wall. Values represent a population of 350 individual cells collected in three independent experimental series. As a rapid indicator of a sensory role for MTs, apoplastic alkalinization (Chang and Nick, 2012) was measured by combining a pH meter (Schott handylab, pH 12) with a pH electrode (Mettler Toledo, LoT 403-M8-S7/120) as described in Qiao *et al.* (2010). Representative time courses from four independent time series are shown.

### Protein extraction and western blot analysis

Protein extracts were prepared according to Jovanovic *et al.* (2010) with minor modifications. After precipitation with trichloracetic acid (Bensadoun and Weinstein, 1976), proteins were dissolved in 125 μl of sample buffer and denatured at 95 °C for 5min. Equal amounts of total protein for the different samples were subjected to SDS–PAGE on 10% (w/v) polyacrylamide gels and subsequently probed by western blotting according to Nick *et al.* (1995) in parallel with a pre-stained size marker (P7709v, New England Biolabs). Tyrosinated α-tubulin was detected by the monoclonal mouse antibody ATT (Sigma-Aldrich; Kreis, 1987), whereas the monoclonal mouse antibody DM1A (Sigma-Aldrich; Breitling and Little, 1986) was used for detection of detyrosinated α-tubulin, DM1A recognizes an epitope localized at amino acids 426–430 of α-tubulin, which is exposed in detyrosinated MTs, but not in tyrosinated tubulin. In a previous study (Wiesler *et al.*, 2002), it could be shown that DM1A does not detect the tyrosinated form of α-tubulin that was readily detected by the antibody ATT. However, when the C-terminal tyrosine was cleaved off by a C-terminal peptidase, the tubulin form that had been ignored by DM1A was bound readily. For signal development, a polyclonal anti-mouse IgG coupled with alkaline phosphatase (Sigma-Aldrich) was employed. Equal loading was verified by running a replicate gel that was stained with Coomassie Brilliant Blue. To quantify the signal, the bands in the western blot were plotted using the plot profile function of ImageJ (NIH, Bethesda, USA), corrected for background, and the integrated density of each band was calculated relative to the value obtained for the control sample. The data represent mean values from two independent experimental series.

### Microscopy

Determination of nuclear position and cross wall orientation of cells was carried out using an AxioImager.Z1 Apotome microscope (Zeiss, Jena, Germany) with a ×20 objective and digital image acquisition controlled by AxioVision Software 4.8 (Zeiss). For examination of the mitotic spindle, confocal *z*-stacks were recorded at day 1 after subcultivation in the AtTUB6–GFP tobacco BY-2 strain using a Zeiss Observer.Z1 microscope with a Yokogawa CSUx1 detection system comprising a ×63 LCI-Neofluar Imm Corr DIC objective (NA 1.3) and the 488nm emission line of the Ar–Kr laser, as well as a spinning-disc device (YOKOGAWA CSU-X1 5000). Analysis of KCH binding on MTs under parthenolide treatment was performed using the same settings. Images were recorded at day 3 after subcultivation under treatment with 10 μM parthenolide. Untreated OsKCH cells at day 3 served as a control.

## Results

### Parthenolide inhibits the growth of cycling BY-2 cells depending on tubulin

**Fig. 1.**
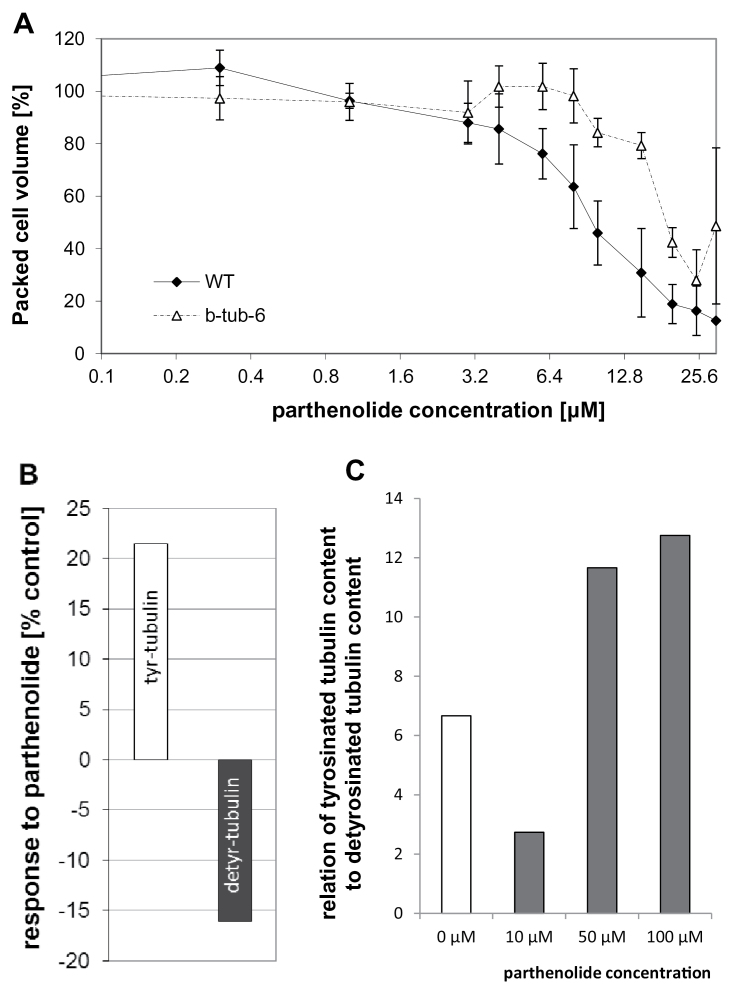
Parthenolide inhibits the growth of cycling BY-2 cells depending on tubulin. (A) Dose–response of the packed cell volume (relative to untreated controls) to parthenolide at the completion of the cycling phase of the culture for non-transformed cells (filled diamonds) and a transgenic cell line overexpressing β-tubulin 6 of *Arabidopsis thaliana* fused to GFP (open triangles). Data show the mean and SEs from measured cell volumes in 15ml cell culture each collected in three independent experimental series. The parthenolide concentration is plotted on a logarithmic scale. (B) Changes in the relative abundance of detyrosinated and tyrosinated tubulin in response to parthenolide as compared with the control without treatment. The abundance of the respective tubulin species relative to the total proteins separated on the gel in the control situation corresponds to 100%. The data represent mean values from two independent experimental series. (C) Relationship of tyrosinated tubulin content to detyrosinated tubulin content dependent on different parthenolide concentrations.

**Fig. 2.**
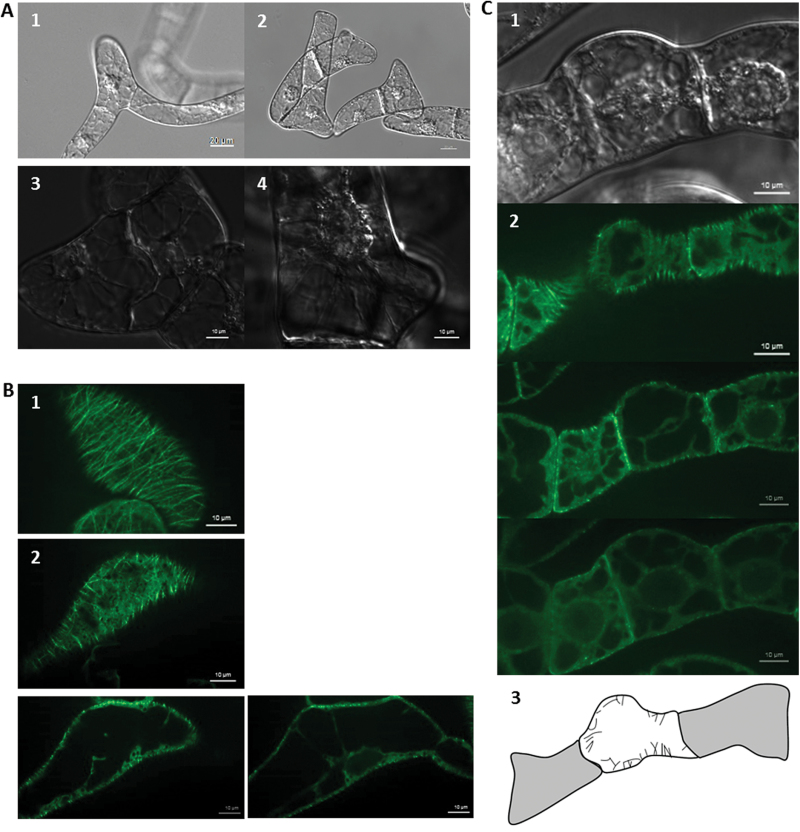
Parthenolide causes a partial loss of axiality correlated with disorganization of cortical microtubules. (A) Morphological aberrations after treatment with 10 μM parthenolide for 4 d. Representative differential interference contrast images of the non-transformed BY-2 wild type. (B) Disorganization of cortical microtubules in the fluorescent tubulin marker line AtTUB6 after treatment with 10 μM parthenolide for 4 d. (1) Confocal section in the cell cortex showing GFP-tagged cortical microtubules of an untreated AtTUB6 control cell. (2) Confocal sections in the cell cortex of a cell treated with 10 μM parthenolide in the GFP filter set. (C) Bulbous swelling of AtTUB6 after treatment with 10 μM parthenolide in the differential interference contrast (1) and the GFP filter set (2). Different confocal sections are shown in the GFP filter set. The schematic representation in (3) highlights the disorientation of the cortical microtubules. (This figure is available in colour at *JXB* online.)

In order to analyse the influence of parthenolide on cycling BY-2 cells, a dose–response curve of packed cell volume as readout was recorded at the completion of the cycling phase, 4 d after subcultivation (Fig. 1A). It was observed that the packed cell volume was significantly decreased by 6 μM of parthenolide, and at 30 μM parthenolide had dropped to 12% of the control. To test whether this inhibition was dependent on tubulin, the dose–response for the transgenic line AtTUB6, overexpressing β-tubulin 6 from *A. thaliana* (Hohenberger *et al.*, 2011), was compared. This line was less sensitive to parthenolide, resulting in a shift of the dose–response curve to ~2-fold higher concentrations, meaning that, compared with the non-transformed line, the concentration of parthenolide had to be doubled to achieve a comparative inhibition. The concentrations of parthenolide used in this dose–response analysis did not affect the viability of cells, as verified by staining with Evan’s Blue (data not shown). However, moderate concentrations of parthenolide (10 μM) which already caused only a partial inhibition of growth (in AtTUB6 even only a very modest inhibition), affected the morphology of cells, both in the non-transformed (Fig. 2A) line and in the AtTUB6 line (Fig. 2B, C). These aberrant morphologies included a loss of polarity in the terminal cells of a file leading to incipient branching (Figs 2A2, 4), as well as lateral swelling of the proximal cells (Fig. 2A1, 2C1), and often were observed in individual cells of a file, whereas the neighbouring cells still appeared relatively normal. Whereas cortical MTs (visualized by the GFP-tagged tubulin) were aligned in parallel arrays perpendicular to the file axis in AtTUB6 cells grown in the absence of parthenolide (Fig. 2B1), microtubular alignment was impaired in those cells exhibiting bulging (Fig. 2B2, C). A comparison of the confocal sections collected from the same stack between the cell cortex and cell centre shows that the effect of parthenolide in the bulging regions is localized in the cell cortex. These findings show (i) that parthenolide inhibits the proliferation of cycling BY-2; (ii) that this inhibition depends on the abundance of tubulin; and (iii) that low concentrations of parthenolide that still allow proliferation already impair the alignment of cortical MTs accompanied by a loss of cellular axiality.

### Parthenolide repartitions tubulin from the detyrosinated to the tyrosinated form

To test for the effect of parthenolide on the tyrosination status of α-tubulin, total extracts from non-transformed BY-2 cells grown either under control conditions or in the presence of parthenolide for 3 d were separated by SDS–PAGE and probed by western blotting using the monoclonal mouse antibodies ATT detecting tyrosinated α-tubulin and DM1A detecting detyrosinated α-tubulin, respectively. To relate the biochemical and physiological effect, different concentrations (0, 10, 50, and 100 μM) were tested for their ability to promote the accumulation of tyrosinated tubulin (Fig. 1C). Since 50 μM was found to be almost saturating, this concentration was then used to test whether the increase of tyrosinated tubulin was accompanied by a corresponding decrease of detyrosinated tubulin: compared with the untreated control, the signal for tyrosinated α-tubulin was elevated by 20% whereas the signal for detyrosinated α-tubulin was reduced by 16%, indicative of a reduced activity of tubulin detyrosination in response to parthenolide (Fig. 1B).

### Parthenolide affects microtubule-dependent events defining division geometry

**Fig. 3.**
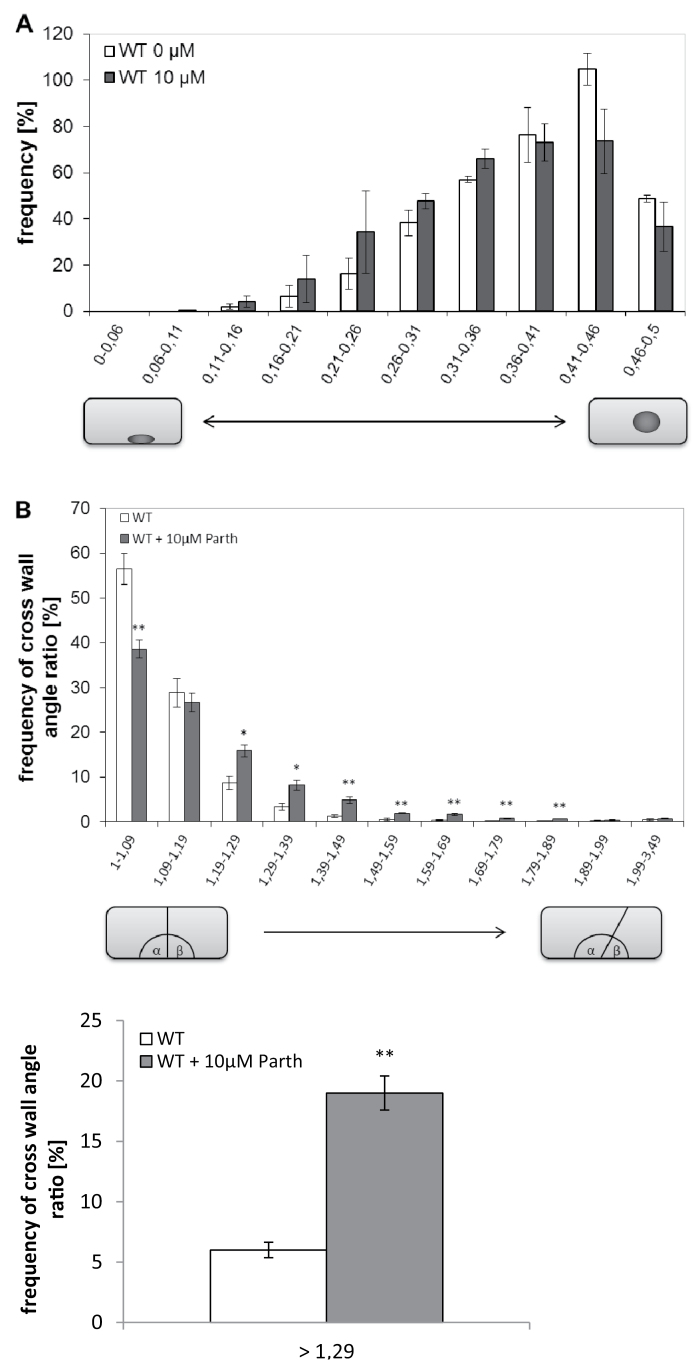
Parthenolide affects cellular events linked to division geometry. (A) Frequency distribution of nuclear positioning at day 1 after subcultivation, recording pre-mitotic nuclear positioning. A value of 0.5 represents completion of the process, when the nucleus has reached the middle of a cell. Before mitosis the nucleus has to be moved towards the middle of the cell. Therefore, a delay in mitosis can be seen as a shift of nuclear positioning with values <0.5. Nuclear positioning was measured at day 1 after subcultivation according to Frey *et al.* (2010). Data show the mean and SEs from 350 individual cells collected in three independent experimental series. (B) Frequency distribution of cross wall orientation (1 represents completely symmetric cross walls). Values represent a population of 350 individual cells collected in three independent experimental series. The shift of the distribution caused by parthenolide is significant at the *P*=95% level. (C) Frequency of cells with disoriented walls (deviation from symmetry ≥30%). The shift of the distribution caused by parthenolide is significant at the *P*=99% level.

MTs play an important role during mitosis. In the cells of higher plants, the division spindle is accompanied by two MT arrays that define the geometry of cell division. The pre-mitotic movement of the nucleus to the cell centre defines the symmetry of division, whereas the post-mitotic formation of the microtubular phragmoplast defines the orientation of the new cross wall (for a recent review, see Nick, 2014). Since both events depend on plant-specific KCH kinesins (Frey *et al.*, 2010), this leads to the question of whether they might depend on detyrosination. Since the response requires some time to become manifest, here a lower concentration of 10 μM parthenolide was used to avoid long-term toxicity. Determination of the frequency distribution during the progress of nuclear positioning (Fig. 3A) showed that this distribution was shifted to smaller values after treatment with parthenolide, demonstrating a delay in nuclear migration towards the cell centre.

**Fig. 4.**
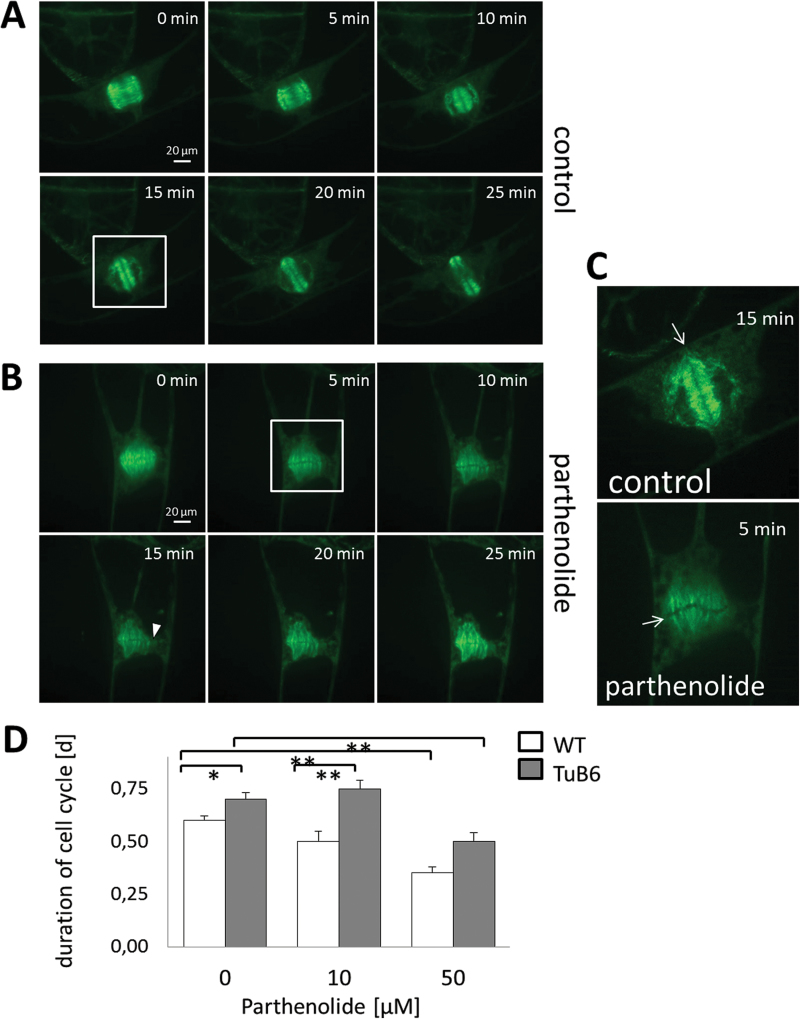
Parthenolide disorients division symmetry. Representative confocal time series of an untreated BY-2 AtTUB6 cell (A) and a cell treated with 100 μM parthenolide (B). (1–6) represent different time points: (1) 0min, (2) 5min, (3) 10min, (4) 15min, (5) 20min, and (6) 25min. (C) Enlargement of the rectangle shown in A and B. Control after 15min and parthenolide treatment after 5min is shown. White arrows indicate the new cell plate. The arrowhead indicates a region of the cell plate that is not lined by phragmoplast microtubules. Note the wavy and partially discontinuous cell plate after parthenolide treatment. (D) Duration of the cell cycle in relation to parthenolide treatment in wild-type and AtTUB6 cells. (This figure is available in colour at *JXB* online.)

When the formation of a phragmoplast was followed over time in untreated AtTUB6 cells (Fig. 4A), the interdigitated MTs in the cell centre delineated a straight darker line. This line delineated by the fluorescent cytoplasm represents the lateral aspect of the fusing cell plate. During mitotic progression, the cell plate expanded in the centrifugal direction. To test the effect of parthenolide on phragmoplast formation, a higher concentration (100 μM) was used, since the time window for inspection by microscopy was short. This showed that in the presence of parthenolide, the two expanding caps of the phragmoplast were not properly aligned, but delineated a wavy and partially non-contiguous cell plate (Fig. 4B). It was also observed that the cell plate in the parthenolide-treated cells partially extended beyond the zone of the phragmoplast MTs (Fig. 4C, white arrows). The question of whether parthenolide, due to its effect on MTs, would slow down the cell cycle was also addressed. However, the opposite was observed: in response to parthenolide, a significant reduction in the duration of the cell cycle is observed for both wild-type and AtTUB6 cells (Fig. 4D). However, this acceleration of the cell cycle is more pronounced in the wild type, but was seen only for the highest concentration in the case of AtTUB6.

Disruption of the phragmoplast is known to interfere with the orientation of the new cross wall. This can be quantified by determining the angle of the cross walls with the lateral walls (Jovanovic *et al.*, 2010). In untreated controls, the orientation of the cross wall was strictly perpendicular to the file axis; in ~60% of the population, the deviation from angular symmetry was <10%, (Fig. 3B) and only 6% of the population showed cross walls that deviated >30% from symmetry. In response to parthenolide, the perpendicular cross walls (deviation from symmetry <10%) dropped to <40%, and strong deviations (>30% from symmetry) increased to almost 20% (Fig. 3C).

Thus, parthenolide specifically affects those microtubular processes that control symmetry (pre-mitotic nuclear migration), or orientation (phragmoplast, cross wall deposition) of cell division. Both events are dependent on the plant-specific KCH kinesin motors.

### Parthenolide increases the association of KCH with cortical microtubules

To visualize the effect of parthenolide on the intracellular distribution of KCH, a transgenic line expressing the rice kinesin OsKCH fused to GFP was used (Frey *et al.*, 2010). In untreated cells, the GFP signal reporting KCH was aligned like beads on a string in characteristic parallel rows transverse to the cell axis (Fig. 6A), with a slow movement of the punctate signals along the row, consistent with the previous observation of a mobile population of KCH associated with cortical MTs (Klotz and Nick, 2012). In response to parthenolide (a lower concentration of 10 μM was used, corresponding to a half-maximal inhibition of growth; see Fig. 1A), the pattern observed was significantly altered (Fig. 6B). Here, the cortical MTs were not decorated by well-separated punctae, but were labelled more continuously (although still interrupted by regions with reduced labelling). Thus, the abundance of GFP signal, which was associated with cortical MTs, was significantly increased in response to parthenolide.

### Parthenolide activates apoplastic alkalinization as a marker for sensory microtubule functions

**Fig. 5.**
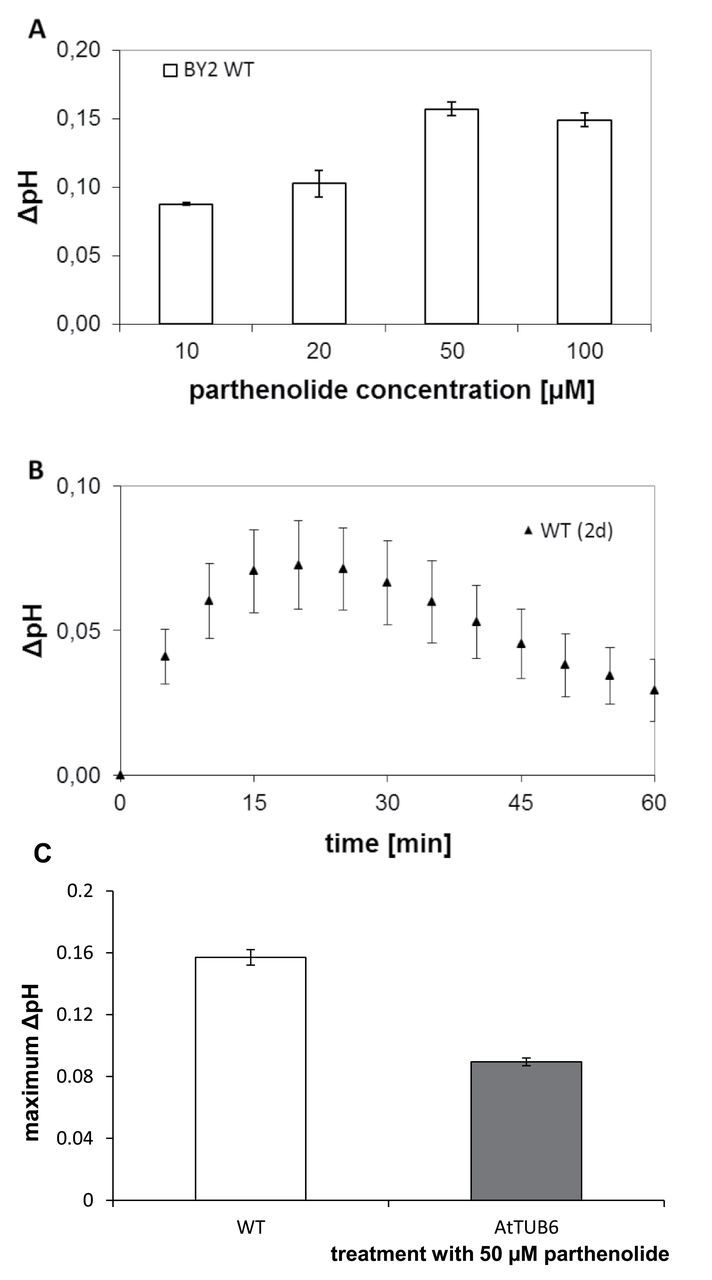
Parthenolide activates apoplastic alkalinization (A) Dose–response curve of maximal alkalinization over different parthenolide concentrations. (B) Representative time course of alkalinization in response to 10 μM parthenolide in cells treated at day 2 after subcultivation. Data represent mean values from four independent experimental series. (C) Maximal alkalinization in response to 50 μM parthenolide in wild-type and AtTUB6 cells at day 3 after subcultivation.

MTs not only define the axiality of plant cells, but also fulfil functions related to the processing of stress-related stimuli. This sensory function is probably linked to the activity of ion channels at the plasma membrane and involves a dynamic subpopulation of MTs (for a review, see Nick, 2013). Stress-related activation of calcium influx channels can be monitored by the simultaneous influx of protons through these channels resulting in an increase of extracellular pH (see, for instance, Guan *et al.*, 2013). Therefore the response of apoplastic alkalinization to parthenolide was followed (Fig. 5B). Upon addition of 10 μM parthenolide, a rapid and transient increase of extracellular pH was already measured at the first measurement time point (5min after addition of parthenolide) and increased further to reach a maximum of ~0.05 pH units at 15min, and subsequently to dissipate slowly. The timing and amplitude of this response were comparable with the response observed when tobacco BY-2 cells are challenged by flg22, a potent activator of basal defence (Guan *et al.*, 2013). By increasing the concentration of parthenolide, the amplitude of the pH peak could be increased up to >0.15 pH units for 50 μM (Fig. 5A) when the response became saturated; a further increase of the parthenolide concentration to 100 μM did not produce further increases in pH. This indicates that the MT population underlying this pH response is small such that its modification is saturated at 50 μM parthenolide. The data might also indicate that this population of MTs is modulating the activity of a calcium influx channel at the plasma membrane. To test whether the response to parthenolide could be outcompeted by excess substrate, the response was also assessed in a transgenic line expressing a GFP fusion of AtTUB6. It was observed that the pH response to 50 μM parthenolide was reduced to about half of that seen in the non-transformed line. Figure 5C shows that the AtTUB6 line has a reduced pH response congruent with the reduced parthenolide sensitivity of cell growth observed in this line (Fig. 1A).

## Discussion

To gain insight into the still elusive biological function of tubulin detyrosination and retyrosination, in a previous study NO_2_Tyr had been used as a strategy to decrease the abundance of detyrosinated tubulin (Jovanovic *et al.*, 2010). During that work, it was possible to detect specific cellular responses such as increased oryzalin sensitivity indicative of stimulated turnover, or misoriented cross walls indicative of affected phragmoplast organization. Although these effects were attributed to the depletion of the detyrosinated form of α-tubulin, they might also be caused by nitro-tyrosinated α-tubulin itself, as shown in Lozano-Juste *et al.* (2011) where potential *in vivo* targets of tyrosine nitration in the *A. thaliana* proteome were analysed based on anti-3-nitrotyrosine antibody and liquid chomatograpy–tandem mass spectrometry (LC-MS/MS). Furthermore nitric oxide (NO), as a key secondary messenger in cells, can target its signal to cytoskeletal proteins via post-translational modifications and mediate tyrosine nitration (Yemets *et al.*, 2011). Here an alternative strategy was therefore tested: using the natural sesquiterpene lactone parthenolide, an inhibitor of TTC activity (Fonrose *et al.*, 2007), it could be shown that the detyrosinated form of α-tubulin can also be down-regulated in plant cells. This is accompanied by dose-dependent inhibition of cell proliferation, a delay of pre-mitotic nuclear positioning, a disturbed geometry of the metaphase plate, and, consequently, a disorientation of the cross walls. These effects are congruent with those reported for NO_2_Tyr (Jovanovic *et al.*, 2010). In addition, rapid apoplastic alkalinization, an early indicator of a calcium influx activity at the plasma membrane that is otherwise observed as an early event during plant defence (Felix *et al.*, 1993), was investigated and it was observed that this sensory event is activated by parthenolide.

The interpretation of cellular responses to inhibitor treatments relies on the specificity of the inhibitor. The following observations support the specificity of parthenolide in the present cellular model. (i) The effect of parthenolide shows a clear dose dependency (Fig. 1) with a half-maximal effect at ~8 µM NO_2_Tyr, which closely matches the dose reported for mammalian cells (Fonrose *et al.*, 2007). (ii) The effect of parthenolide is shifted to higher doses in a cell line where MTs, due to overexpression of GFP–TUB6, are stabilized (Fig. 1), parallel with a reduced parthenolide response of apoplastic alkalinization in this line. (iii) The cellular responses to parthenolide are congruent, even with respect to all their details, with the responses observed for NO_2_Tyr (Jovanovic *et al.*, 2010), although the molecular target of the two compounds is different, supporting the conclusion that it is the reduction in detyrosinated α-tubulin that mediates the cellular effect.

**Fig. 6.**
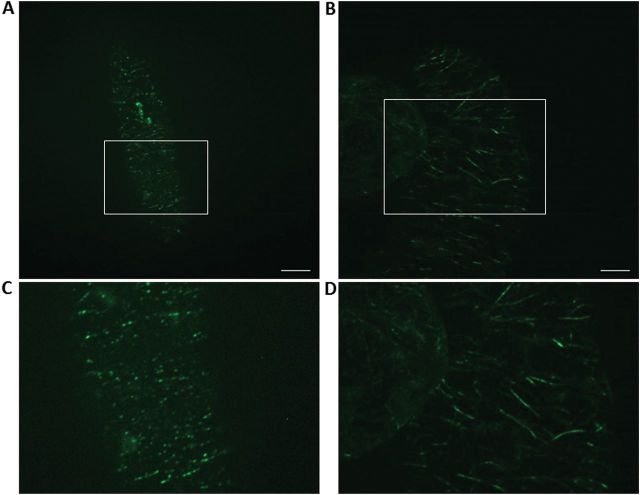
Parthenolide recruits the plant-specific kinesin KCH to cortical microtubules. Representative images show the localization of a KCH–GFP fusion in an untreated control cell (A), and a cell treated with 10 μM parthenolide for 3 d (B). (C, D) Enlargement of the rectangle shown in A and B. (This figure is available in colour at *JXB* online.)

For these reasons, the cellular response to parthenolide can be attributed to the reduced level of detyrosinated α-tubulin. Detyrosinated α-tubulin is preferentially found in stable MTs, and this led to the hypothesis that detyrosination might confer stability to a given MT (Schulze *et al.*, 1987). Alternatively, detyrosination might just be the consequence of stability (Khawaja *et al.*, 1988). Since MTs assemble and disassemble in the manner of a treadmill, a tubulin heterodimer integrated at the plus-end of the MT will progressively travel towards the minus-end and eventually leave the MT. The duration of this journey depends on the velocity of the treadmill, which can be quite diverse in different MT populations. Since TTL preferentially binds non-assembled heterodimers of tubulin (Burns, 1987), whereas TTC prefers assembled MTs (Gundersen *et al.*, 1987), stable MTs would be detyrosinated in preference, whereas dynamic MTs would escape the activity of TTC. This would create a code, recruiting stable microtubules as targets for the binding of MT-associated proteins (MAPs) (Janke and Kneussel, 2010; Wloga and Gaertig, 2010) with higher affinity for modified MTs (Verhey and Gaertig, 2007). In fact, several kinesins have been shown to bind predominantly to detyrosinated MTs (Liao and Gundersen, 1998; Zekert and Fischer, 2009). However, the link between detyrosination and stability might also be bidirectional: some stabilizing MAPs were found to bind more easily to detyrosinated microtubules (de Forges *et al.*, 2012), and binding of the MCAK of mammalian cells is even inhibited by detyrosination (Peris *et al.*, 2009). Thus, the specific decoration of a given MT by a kinesin will depend on the type of kinesin. The observation that the punctate decoration of cortical MTs by KCH is transformed into a more continuous label after treatment with parthenolide (Fig. 6) indicates that this plant-specific kinesin is of the type preferring tyrosinated microtubules. That detyrosination or tyrosination of α-tubulin defines the affinity of kinesins towards MTs is accompanied by the fact that nitration of tubulin also plays a role in interaction of kinesins with MTs. The interaction of kinesin-8, for example, is not so strong with detyrosinated tubulin as with tubulin nitrated polymers. Kinesin-1 instead prefers binding to detyrosinated MTs. (Blume *et al.*, 2013)

The current study shows that parthenolide specifically affects microtubular processes in plants that control either symmetry (pre-mitotic nuclear migration) or orientation (phragmoplast and cross wall deposition) of cell division. Both events are dependent on plant-specific kinesin motors: nuclear migration is controlled by actin filaments and MTs around the nucleus forming a perinuclear cage linked by KCH, a plant-specific kinesin with a calponin homology domain (Klotz and Nick, 2012). This kinesin occurs in two subpopulations; while it is tethered to actin around the nucleus, it is detached from actin in the cell cortex (as well as in the phragmoplast). The pre-mitotic nuclear migration defines the symmetry of cell division and is followed by intensive remodelling of the microtubular cytoskeleton constituting the division spindle. The subsequent array, the phragmoplast, is laid down at the site where the preprophase band had persisted prior to mitosis. Classical experiments in fern protonemata by Murata and Wada (1991), where the nucleus was separated from the pre-prophase band by centrifugation, demonstrated that the nucleus does not require a pre-prophase band to complete mitosis. However, the absence of the pre-prophase band impairs the generation of the new cell plate that becomes disoriented or even incomplete. The guiding effect of the pre-prophase band on the spatial organization of the phragmoplast and cell plate seems to be caused by MT-dependent transport in the pre-prophase band: endosomal vesicles move along the MTs of the pre-prophase band and are laid down in a belt around the prospective division plane. This endosomal belt is later (after mitosis has been completed) read out by exploratory MTs constituting the phragmoplast (for a review, see Nick, 2012). The set-up of the mitotic spindle involves massive nucleation and elongation of novel MTs, and thus depends on the affinity of end-binding proteins. While some kinesins prefer detyrosinated tubulin, the plus-end proteins show a higher affinity for tyrosinated tubulin (for a review, see Cai, 2010). This makes sense, since the dynamic plus-end of an MT is tyrosinated to a higher extent as compared with the rest of the MT. Inhibition of detyrosination due to parthenolide treatment is expected to generate a longer tyrosinated domain at the plus-end, which allows KCH with its apparently higher affinity for tyrosinated tubulin to bind to the basal domains of cortical MTs, where it otherwise would not be able to bind. This model might also explain why KCH moves by a factor of 2–3 times more slowlyr as compared with other plant kinesins (Klotz and Nick, 2012). The low value of only 3.2 μm min^–1^ closely matches the 3.5 μm min^–1^ of the growing plus-end of cortical MTs (Shaw *et al.*, 2003). The most straightforward explanation would be that KCH binds to the tyrosinated plus-end of cortical MTs and translocates through treadmilling of the MT. When the microtubular-binding domain is extended as a consequence of parthenolide treatment, this will recruit more KCH to the cortical MTs and interfere with microtubular directionality by ectopic cross-linking with actin filaments. This might explain the observed loss of MT orientation and the cell axis (Fig. 2). Conversely, parthenolide treatment is expected to increase the decoration of the perinuclear MTs with KCH, which should cause a tighter cross-linking with actin, increasing the rigidity of nuclear tethering. Parthenolide would thus cause a functional phenocopy of KCH overexpression. In fact, overexpression of KCH delays pre-mitotic nuclear migration in a similar manner to parthenolide treatment (Frey *et al.*, 2010). The excessive tethering of MTs with actin filaments is also expected to impair the deposition of the endosomal belt subtending the microtubular pre-prophase band, which should cause disorientation of the cell plate. This is what was observed in this study. The wavy appearance of the cell plate after treatment with parthenolide correlates with a situation where the cell plate in the parthenolide-treated cells partially extends beyond the zone of the phragmoplast MTs. Interestingly, despite the fact that parthenolide impairs the formation of the cell plate, it does not seem to delay the cell cycle, but instead reduces the length of the cell cycle in a concentration-dependent manner. This acceleration is significantly dampened in the AtTUB6 line, indicating that the additional tubulin dimers can titrate the effect of the drug. Moreover, the reduction of the duration of the cell cycle in response to parthenolide indicates that the abundance of detyrosinated tubulin might be a factor that is limiting cell cycle progression.

In addition to cell division, kinesins are known to act in the cortical MT array underneath the plasma membrane (for a review, see Cai and Cresti, 2012). These cortical MTs not only control the axiality of cell expansion by guiding the movement of cellulose-synthesizing enzyme complexes in the plasma membrane and thus influencing the texture of the cell wall (for a recent review, see Nick, 2014), but, in addition, are also important components of a signalling hub central to the processing of abiotic stress and can, specifically, sense and integrate the mechanical load in the cell wall (for a review, see Nick, 2013). Detyrosination seems also to be relevant for the sensory function mentioned above: parthenolide triggers a dose-dependent activation of apoplastic alkalinization indicative of a calcium influx channel involved in defence (Felix *et al.*, 1993). Whether this phenomenon is caused by the enhanced binding of KCH remains to be elucidated. The fact that the parthenolide effect can be partially outcompeted by offering excess tubulin dimers (using the AtTUB6 line) is evidence for a direct role for MTs in this process. At this stage, it should be noted that the present observations are congruent with patch clamp studies on calcium channels residing in the plasma membrane that have shown a clear dependence on MTs (Ding and Pickard, 1993; Mazars *et al.*, 1997). The elevated recruitment of KCH to the plus-end might also interfere with the binding of end-binding proteins—it should be mentioned here that loss-of-function mutants for EB1 in *Arabidopsis* are strongly impaired in the sensing of touch and gravity stimuli (Bisgrove *et al.*, 2008).

### Outlook

The current work confirms the findings by Jovanovic *et al.* (2010) using a different strategy to reduce the abundance of detyrosinated α-tubulin in the BY-2 system. Since TTC has remained elusive (Ersfeld *et al.*, 1993), so far the only strategy to modulate detyrosination through genetic engineering was to use TTL as the target. A tobacco cell line overexpressing a putative TTL from rice under control of a constitutive promotor fused to red fluorescent protein was therefore generated. To obtain insight into the physiological function of the detyrosination cycle, it is necessary to move from the cell culture into real plants. Rice plants that have the same rice TTL construct in the homologous system therefore also been generated and currently the phenotypical and developmental consequences of TTL overexpression are under investigation.
